# Acute Metabolic Changes with Thigh-Positioned Wearable Resistances during Submaximal Running in Endurance-Trained Runners

**DOI:** 10.3390/sports7080187

**Published:** 2019-08-01

**Authors:** Allister P. Field, Nicholas Gill, Paul Macadam, Dan Plews

**Affiliations:** 1 Sports Performance Research Institute New Zealand (SPRINZ) at AUT Millennium, Auckland University of Technology, Auckland 0632, New Zealand; 2 Adams Centre for High Performance, University of Waikato, Tauranga 3116, New Zealand

**Keywords:** limb loading, heart rate, oxygen consumption

## Abstract

The aim of this study was to determine the acute metabolic effects of different magnitudes of wearable resistance (WR) attached to the thigh during submaximal running. Twenty endurance-trained runners (40.8 ± 8.2 years, 1.77 ± 0.7 m, 75.4 ± 9.2 kg) completed six submaximal eight-minute running trials unloaded and with WRs of 1%, 2%, 3%, 4% and 5% body mass (BM), in a random order. The use of a WR resulted in a 1.6 ± 0.6% increase in oxygen consumption (VO_2_) for every 1% BM of additional load. Inferential based analysis found that the loading of ≥3% BM was needed to elicit any substantial responses in VO_2_, with an increase that was likely to be moderate in scale (effect size (ES) ± 90% confidential interval (CI): 0.24 ± 0.07). Using heart rate data, a training load score was extrapolated to quantify the amount of internal stress. For every 1% BM of WR, there is an extra 0.17 ± 0.06 estimated increase in training load. A WR ≥3% of BM was needed to elicit substantial responses in lactate production, with an increase which was very likely to be large in scale (ES ± 90% CI: 0.41 ± 0.18). A thigh-positioned WR provides a running-specific overload with loads ≥3% BM, resulting in substantial changes in metabolic responses.

## 1. Introduction

Endurance running attracts millions of participants both recreationally and competitively across the globe. Modifiable factors, such as training, play a vital role in enhancing the qualities that determine running performance. The physiological mechanisms that determine endurance running performance include the maximum volume of oxygen that can be ventilated, delivered and used by the body’s cells at sea level (VO_2_max) [1], the percentage of VO_2_max that a runner can sustain before blood lactate accumulation exceeds clearance (%VO_2_max at second ventilatory threshold VT_2_) [2,3], and the metabolic cost of running at a given velocity (RE) [1]. Many trained runners also use resistance training to improve their running performance, be it by indirectly improving RE, muscular power capabilities or running performance [4]. Moreover, in recreational distance runners, heavy resistance, explosive resistance and muscle endurance resistance training have been found to significantly improve running performance [5]. It is therefore the objective of the practitioner to fully understand the appropriate training determinants and to be able to effectively and efficiently program training, ensuring the optimal transfer to performance and the minimization of injuries. At elite levels, the concepts of progressive overload and specificity become more important. 

One means of progressive overload is through wearable resistance (WR), which involves an external load being attached to areas of the body enabling a sport-specific form of loading [6,7]. Previous studies have used a wide magnitude of loads (<1% to 40% body mass (BM)) attached to the trunk or limbs during different sporting actions [6,8]. Though heavier loads (>10% BM) have been used with trunk WR, lighter WR loads (<8.5% BM) have been sufficient to significantly increase metabolic demands compared to unloaded walking and running, indicated by increases in oxygen consumption (VO_2_), heart rate (HR), energy workload and energy cost [6]. With metabolic demands being greater with lower body limb loading and demands increasing as comparable load is moved to a more distal position [9,10,11], Martin [11] found that adding 0.25 (0.69% BM) and 0.50 kg (1.39% BM) to each thigh at a running velocity of 12 km·h^−1^ increased VO_2_ by 1.7% and 3.5%, respectively. The researchers noted that HR increases were consistent with increases in VO_2_, but also noted that HR was less sensitive to lower extremity loading [11]. The WR loads used by Martin [11] were added via packets of lead pellets, which were sown into pockets within the shorts. Similarly, the majority of the previous WR running research that has investigated metabolic cost involved cumbersome methods for attaching the external load, which may have unfavourably affected the running mechanics [6]. Moreover, limited research into endurance runners and even more so trained endurance runners has been completed. It is also unknown how a spectrum of incremental WR loads will impact metabolic cost and what this relationship looks like. Understanding the metabolic effects from between loads may enable practitioners to prescribe a more appropriate loading %BM to target different aspects of training. Given the improved technology in recent WR loading, and the fact that an incremental spectrum of loading has yet to be investigated, the purpose of this study was to investigate how a magnitude of between 1% and 5% BM WR attached to the thigh affected the acute metabolic responses to submaximal running in endurance-trained runners. It was hypothesized that the additional loading from the WR would overload the leg musculature, resulting in greater metabolic responses during submaximal running. 

## 2. Materials and Methods

### 2.1. Subjects

Twenty endurance-trained runners (two female and 18 male, 40.8 ± 8.2 years, 1.77 ± 0.7 m, 75.4 ± 9.2 kg) were recruited for the current study. All of the runners had no history of any major health issues and had completed a minimum of one half-marathon distance event in the 12 months prior to the commencement of the study. In addition, they were required to be actively engaged in endurance running training at the onset of the study and have an average VO_2_max of 59.6 ± 7.9 mL·kg^−1^·min^−1^. The ethical approval for this study was obtained from the AUT University Ethics Committee. Before testing, all of the participants provided their informed consent in writing and completed a pre-exercise health questionnaire (Par-Q).

### 2.2. Procedure

**Figure 1:**
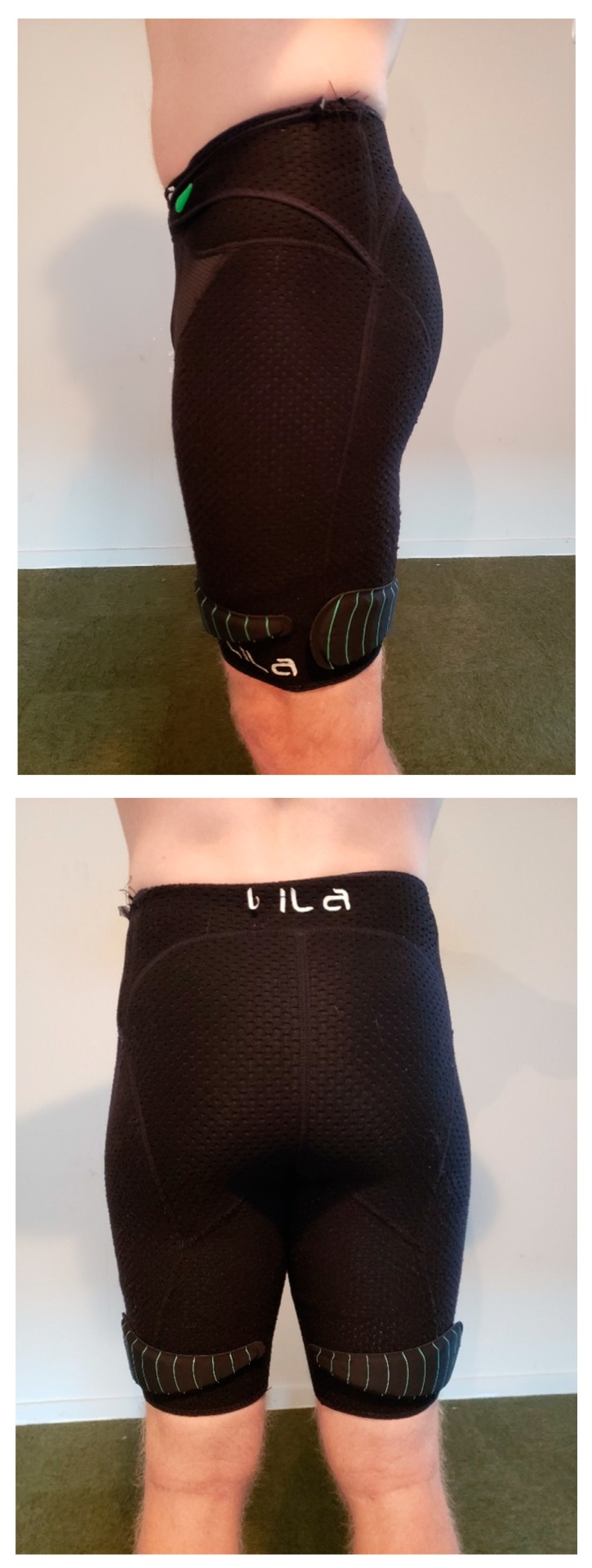
Example of a thigh wearable resistance loading pattern (1% body mass).

**Figure 2:**
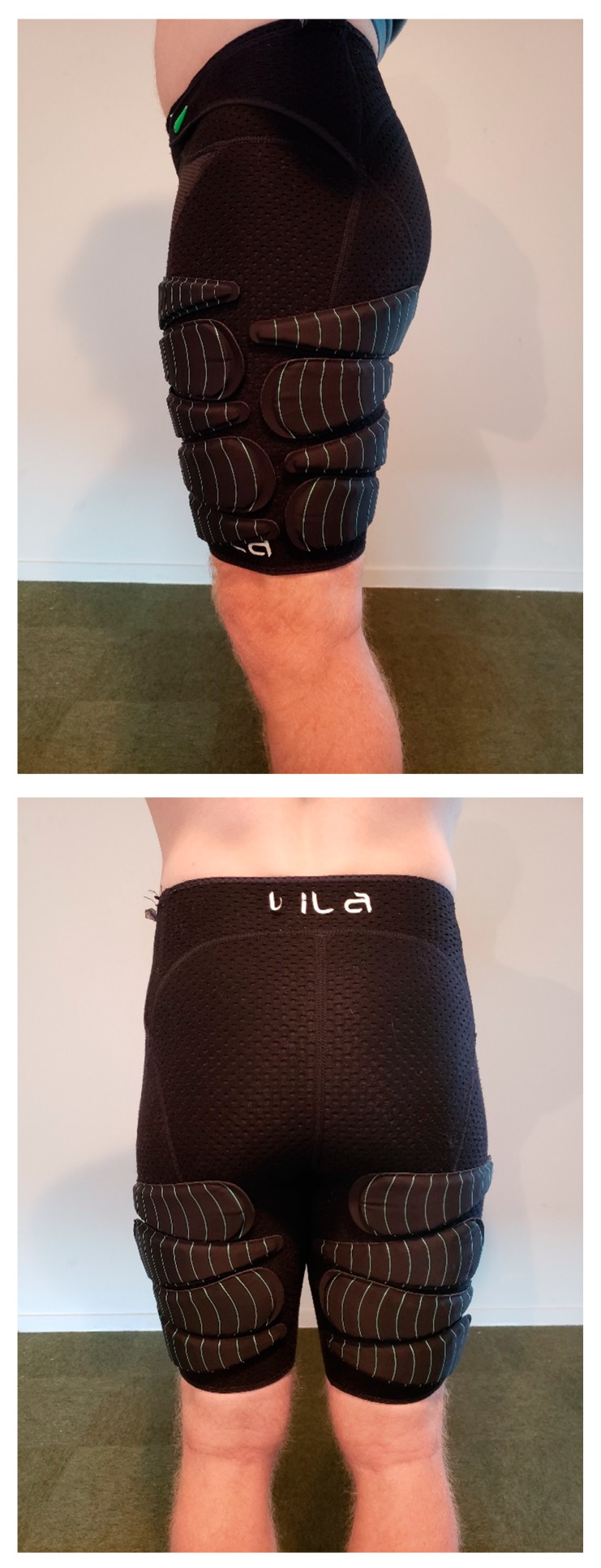
Example of a thigh wearable resistance loading pattern (5% body mass).

All running trials were conducted under stable laboratory conditions (ambient conditions: 21 ± 3 °C, <60% relative humidity) on a motorized treadmill (Woodway, Waukesha, WI, USA) with the gradient set a 1% [12]. The HR response data was collected using an HR monitor (Polar A300, China) and the oxygen consumption data was measured using a carbon dioxide and oxygen analyzer (Metalyzer Cortex, Biophysik GmbH, Leipzig, Germany), which was calibrated before each testing session according to the manufacturer’s specifications. All of the capillary samples were drawn from the preferred finger of the runner and lactate (LA) accumulation was measured using a blood LA analyzer (LA Pro 2, Shiga, Japan). Any subjective data was measured by way of the rate of perceived exertion (RPE) using a modified BORG 10-point scale [13]. For the WR conditions, subjects were required to wear a pair of compression shorts with associated loads (Lila^TM^, Exogen^TM^, Wilayah Persekutuan Kuala Lumpur, Malaysia). The WR was added via 100 or 200 g increments and the total load for each trial was rounded to the nearest 100 g. The loading schemes began with each WR load placed alternatively from the anterior to the posterior, in a stacked balance of distal to proximal (see Figure 1 and Figure 2). A spectrum of WR loading was used (0%, 1%, 2%, 3%, 4%, and 5% BM) which was comparable to previous WR studies [11,14,15,16,17], to examine changes between the loading magnitudes. 

**Figure 3:**
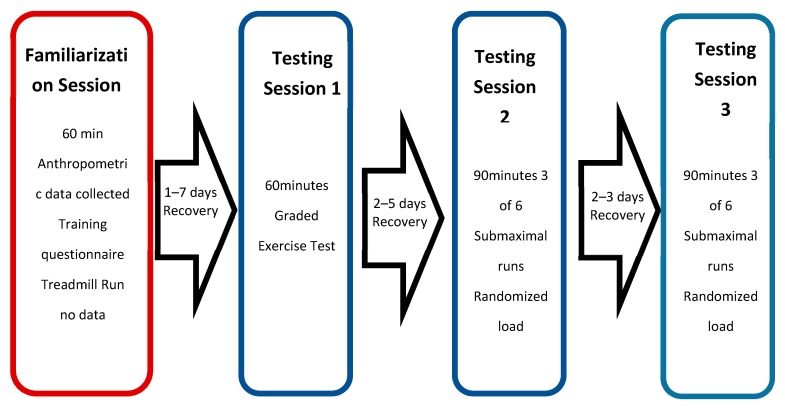
Structure of the testing sessions.

For each participant, the study was conducted over a maximum of 15 days. This included one familiarization session and three testing sessions (see Figure 3) under laboratory conditions. The purpose of the familiarization session was to allow each runner to become accustomed to treadmill running while wearing both the compression shorts and all the metabolic measuring equipment. The participants completed a self-paced run for 20 min followed by a 10 min recovery. During this recovery time, the graded exercise test (GXT) protocol was discussed and an HR monitor and gas mask fitted. The participants then completed a further 10 min run—including a sufficient amount of the GXT incremental protocol, to feel comfortable with the procedures—and no data was collected. The participants were instructed to refrain from training on the day of testing session one and to avoid any strenuous training sessions 24 h prior.

Testing session one occurred within seven days of completing the familiarization session. The purpose was to generate a VO_2_ response profile to graded exercise to establish VT_1_, VT_2_ and VO_2_max. The participants completed a self-paced 20 min warm-up on a treadmill and were given a recovery period of 10 min prior to the commencement of the GXT. The starting speed was maintained for 1 min, followed by an increase of 0.5 km·h^−1^ every 30 s until the point of voluntary exhaustion [18]. The starting speed was adjusted on an individual basis, to ensure volitional exhaustion at between 8 and 12 min. VO_2_ was tracked continuously at a sampling rate of 0.1 Hz, and the HR and RPE were recorded at each speed increment, with LA being measured immediately after completion of the test. The maximum oxygen consumption was, on average, over 30 s and was considered to be achieved if any one of the following criteria were met: a plateau in VO_2_ was reached, despite an increase in workload; a respiratory exchange ratio (RER) >1.15 was observed; an HR within five beats of the age predicted maximum (220-AGE) was reached; or a peak exercise blood LA concentration >8 mmol/L was achieved [19]. The testing sessions two and three included all submaximal running trials, to measure metabolic and subjective responses while un-loaded and loaded. Testing session two occurred within 2–5 days of testing session one and testing session three occurred within 2–3 days of testing session two, to ensure no fatigue between all three sessions (Barnett, 2006). Testing session two included three WR loads, testing session three included the final three WR loads and load order was randomized. At the start of both testing sessions two and three, an 8 min warm up—set at a running speed equivalent to VT_1_—was completed, followed by a 10 min recovery. VT_1_ was chosen as this is close to the typical training intensity in endurance sports, in line with the polarized model of training. The polarized training model has been shown to be common practice among elite endurance runners, for whom long, slow distance training at lower intensities (<VT_2_) makes up 75% of an individual’s training volume, with shorter, higher intensity bouts of effort (>VT_2_) making up the remainder of the training program [13]. Each submaximal running trial lasted 8 min, with 10 min seated recovery between each subsequent trial. The oxygen consumption and HR were tracked for 2 min prior to each trial starting, for the 8 min of each trial (with the final 2 min used for analysis) and for 2 min post trial. The rate of perceived exertion and LA was recorded immediately post completion of each 8 min trial.

#### Statistical Analysis

Descriptive statistics—including the means and standard deviations—were calculated for each measure. The statistical aim of this study was to make an inference about the impact on metabolic stress of submaximal running with WR, which requires a determination of the magnitude of an outcome. The traditional sample size estimation and hypothesis testing approach was not appropriate for this study design [20]. Accordingly, inferential statistics were used to examine the qualitative meaning of the observed changes in the metabolic cost (VO_2_, HR, LA) and perception (RPE) of submaximal running, with loaded compared to unloaded examples. The collected data was presented as the mean value for each, with the reported effect size (ES) and percent differences at a 90% confidence interval (CI). The smallest worthwhile change was used to determine if any observed changes were considered trivial, possible or likely, including the magnitude of each change, calculated as a change in score standardized to 0.2 of the between–subject SD from the unloaded condition [21]. The qualitative probabilities were defined by the scale <0.5% most likely trivial increase, <5% very likely trivial increase, <25% likely trivial increase, 25–75% possible small increase, >75% likely moderate increase, >95% very likely large increase, >99.5% most likely very large increase and the outcome was deemed unclear where the 5% and 90% CI of the mean change overlapped with both the positive and negative outcomes [20]. To help quantify the metabolic cost of WR based on relative exercise intensity and duration, HRs were used to extrapolate a training load score (TLS) for each load [22] for 10 min of running. To understand the relationship between metabolic variables (VO_2_, HR and TLS) and load, a scatterplot was created in excel to establish a linear equation and R^2^ value for each variable. The formula used for calculating the Training Load Score (Training Stress Score (TSS) [22] was:

TLS = (sec × HR × IF)/(VT_2_ × 3600) × 100 IF (intensity factor) = HR/VT_2_

Key: TLS: Training load score, HR: Heart rate (average heart rate during exercise), IF: Intensity factor, VT_2_: Second ventilatory threshold (the point at which LA accumulation exceeds clearance). 

## 3. Results

**Figure 4:**
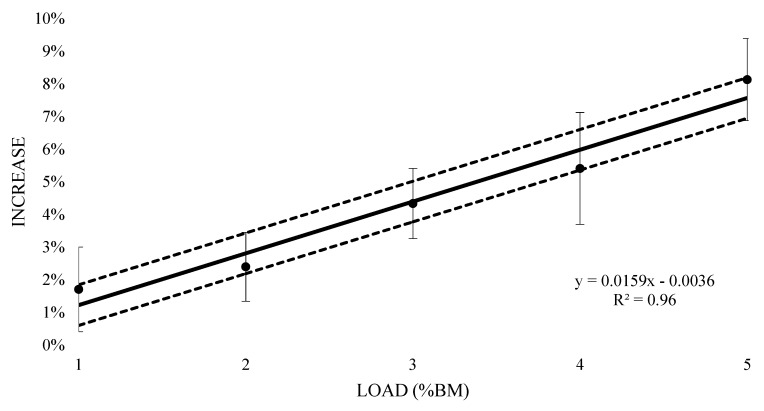
Percent increase in acute oxygen consumption with thigh wearable resistances, compared to unloaded 8 min submaximal running trials.

The mean oxygen consumption of submaximal running at 1% BM was 3.67 L (±0.59) with an increase of 1.7% (±0.01), however this resulted in a likely trivial increase (0.13 ± 0.08) (Table 1). Similarly, a very likely trivial increase was witnessed at 2% BM (0.06 ± 0.7) with a mean oxygen consumption of 3.73 L (±0.62) and a 2.4% (±0.01) increase. Both 3% and 4% BM reported a likely moderate increase (0.24 ± 0.07 and 0.29 ± 0.09 respectively), with mean oxygen consumption values of 3.80 L (±0.62) and 3.84 L (±0.64) respectively, and 4.3% (±0.01) and 5.4% (±0.02) increases respectively. The 5% BM condition saw a most likely very large increase (0.43 ± 0.07) at 3.94 L (±0.66) mean oxygen cost and an 8.1% (±0.01) increase. Figure 4 contains the percentage change in oxygen response from unloaded to loaded (±90% CI). Linear regression was carried out and showed a positive relationship (R^2^ = 0.96), representing an additional 1.59% (±0.62%) increase in oxygen consumption for every 1% BM of additional load. 

**Figure 5:**
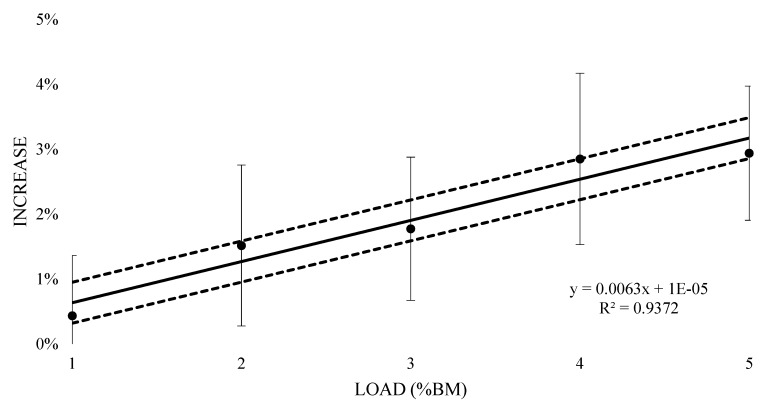
Percent increase in the acute heart rate response to thigh wearable resistance compared to unloaded 8 min submaximal running trials.

**Figure 6:**
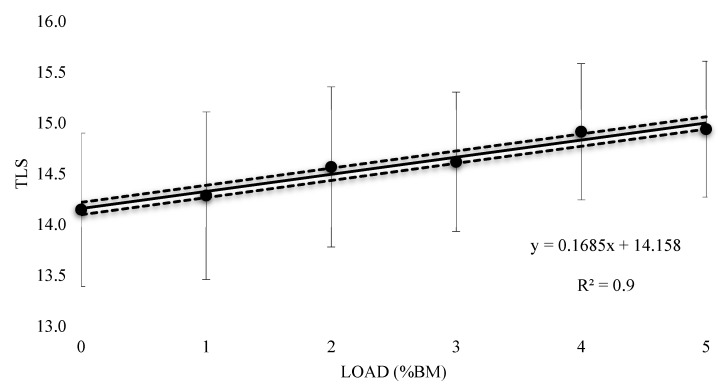
Extrapolated Training Load Score (TLS) for thigh wearable resistance for the equivalent of 10 min of running.

The mean HR response to submaximal running with a load of 1%BM was 158 bpm (±13.42), with a 0.4% (±0.01) increase and resulted in a very likely trivial increase (0.05 ± 0.11) (Table 2). A Possible small increase was witnessed at 2% and 3% BM (0.17 ± 0.15 and 0.2 ± 0.13 respectively), with mean values of 159.50 (±13.42) and 160 bpm (±12.35) respectively, with 1.5% (±0.01) and 1.8% (±0.01) increases respectively. There was a mean HR response of 162 bpm (±11.99) and a 2.9% (±0.01) increase at 4% BM, reporting a likely moderate increase (0.32 ± 0.16). At 5% BM, a very likely large increase (0.33 ± 0.12) with a mean HR response of 162 bpm (±11.36) and 2.9% (±0.01) increase was seen. Figure 5 contains the percentage change in HR responses from unloaded to loaded (±90% CI). Linear regression was carried out and showed a positive relationship (R^2^ = 0.94), representing an additional 0.63% (±0.32) increase in HR response for every 1% BM of additional load. Figure 6 represents the relationship between the TLS extrapolated from the HR data for the equivalent of 10 min of running at VT_1_ and load. The regression equation showed a positive linear relationship (R^2^ = 0.96), representing an additional 0.17 (±0.06) of internal training stress for every 1% BM of additional load for 10 min of running.

The blood LA responses post submaximal running with a load of 1%BM resulted in a mean accumulation of 2.77 mmol/L (±1.90), however, an unclear effect was exhibited with more data needed (0.0 ± 0.28) (Table 3). A likely trivial increase at 2% BM (0.08 ± 0.15) with a mean accumulation of 4.83 mmol/L (±2.04) was observed. The loads at 3% and 4% BM reported very likely large increases (0.41 ± 0.18 and 0.42 ± 0.19 respectively) with mean accumulations of 3.27 (±1.79) and 3.30 mmol/L (±2.03) respectively. Loads at 5% BM produced a mean accumulation of 3.52 mmol/L (±2.35) and reported a most likely very large increase (0.49 ± 0.15).

Post submaximal running with a load of 1% BM resulted in a possible small increase (0.28 ± 0.25) and a mean reported score of 3.35 (±1.16) in RPE (Table 4). There was a likely moderate increase at 2% BM (0.43 ± 0.23), with a mean reported score of 3.68 (±1.44) and there was a mean reported score of 3.73 (±1.33) and very likely large increase at 3% BM (0.52 ± 0.26). Both 4% and 5% BM reported most likely very large increases (0.82 ± 0.29 and 0.86 ± 0.28 respectively), with mean reported scores of 4.20 (±1.26) and 4.38 (±1.57) respectively.

## 4. Discussion

The aim of this study was to understand the acute metabolic effects of thigh WRs during submaximal running in endurance-trained runners. It was found that for every 1% BM of additional load, there is an expected 1.59% (±0.62) and 0.63% (±0.32) increase in VO_2_ and HR response respectively. A thigh WR of at least 3% BM was needed to have a likely moderate increase (0.24 ± 0.07) in VO_2_ response, with a most likely very large increase (0.43 ± 0.07) exhibited at 5% BM. A loading of at least 2% BM was needed to have a possible small increase (0.17 ± 0.15) in HR response, with a very likely large increase (0.33 ± 0.12) at 5% BM. This resulted in a predicted 0.17% (±0.06) increase in internal stress for every 1% BM of additional load for 10 min of running at a speed equivalent to VT_1_. For the mean RPE, a loading of 2% resulted in likely moderate increases, with 3% BM needed for very likely large increases. Findings from this study provide further WR information for practitioners over a magnitude of thigh WR loads, to effectively integrate WR into their training regimes.

The VO_2_ and HR data collected in this study agrees with previously reported findings, showing that limb loading during locomotion can increase the metabolic cost compared to unloaded [9,10], however, these studies investigated lower extremity loading (feet). Martin’s [11] is the only other metabolic study to use thigh loading, and reported an increase in oxygen consumption of 1.7% and 3.5% when the equivalents of 0.69% and 1.39% BM, respectively, were added to the thighs of highly trained male distance runners, with increases in VO_2_ response to load reaching statistical significance (*p* < 05). From the findings in this study, the incremental loading of 1% to 5% BM resulted in linear increases in VO_2_ from 1.7% to 8.1%. Comparatively, an increase in VO_2_ of 1.59% for every 1% BM (equivalent to 0.75 kg when extrapolated from the mean weight of our participants) of additional load was also observed. Accordingly, the increase in cost is slightly less compared to the findings of Martin [11]. The statistical method used in the current study (inferential based analysis), demonstrated that thigh WR loading of at least 3% BM was needed to lead to a likely moderate increase (0.24 ± 0.07). Martin [11] produced statistically significant increases in oxygen consumption at loads lower than 3% BM on the thighs, however, they did not report any effect sizes to establish the magnitude of this change. From studies that used heavier WR attached to the trunk, non-significant, small effect size increases (0.1%–0.3%) in VO_2_ were found during 3 min running at four speeds (9.6–13.1 km/h) utilizing 5% and 10% BM loading [23], while moderate and significant increases (6%–17%) were found during walking with heavier WR (10%–20% BM) from 4 min stages at speeds of 3–6.4 km/h [24]. Therefore—despite the lighter loads used in this study (1%–5% BM)—when WR is attached to the thighs, greater VO_2_ changes occur compared to trunk placement, most likely due to the greater inertial demands from distal thigh loading. 

In terms of HR responses, Martin [11] only reported on mean values, but showed a similar trend to that of VO_2_ in that HRs increased slightly with additional loads to the thighs. These changes, however, did not reach statistical significance and the researchers suggested that HR is a less sensitive measure of thigh loading under 1.39% BM. Comparatively, in this study, an increase in HR of 0.63% was found for every 1% BM (equivalent to 0.75 kg when extrapolated from the mean weight of our participants) of additional load, which is less than half that of VO_2_ (1.59%) for the same load. Inferential based analysis demonstrated that thigh loading of at least 2% BM was needed to lead to a possible small increase (0.17 ± 0.15) in HR response with 1% BM reporting a very likely trivial increase (0.05 ± 0.11). Using the HR data collected, a TLS was extrapolated to help quantify the amount of internal stress each loaded trial would have over a 10 min running period. Based on the linear regression equation produced for TLS plotted against load, for every 1% BM of additional load, there is an extra 0.17 increase in internal stress. A TLS was calculated, as it is a commonly used method for quantifying training stress for endurance athletes and may help put into context the impact that added load has on HR. Linear increases were found in mean RPE (3.08 to 4.38) with a loading of 2%, resulting in likely moderate increases, while very likely large increases were found with 3% BM. The authors note and acknowledge that the following limitations of the research should be considered for future investigations. This study may only suggest the possible responses found during the repeated application of such loading schemes to short-term submaximal running at a speed equivalent to VT_1_. Due to the subject inclusion criteria (male and female endurance-trained runners), the findings of this study may only be applied to this population. Trials were performed under laboratory conditions, which are not directly comparable to the traditional method of training for endurance-trained runners. 

## 5. Conclusions

The current findings suggest that using thigh-loaded WRs while running at a speed equivalent to VT_1_ will elicit an increase in metabolic response when compared to unloaded running. There is an expected increase in VO_2_ and HR response of 1.59% (±0.62) and 0.63% (±0.32), respectively, for every 1% BM of additional load and an increase in exercise stress of 0.17 (±0.06) for the equivalent of 10 min of running for every 1% BM of additional load. However, loads of at least 2% and 3% BM are needed to see substantial increases in HR and VO_2_ responses respectively. The findings from the current study provide some evidence for quantifying the potential increases in both VO_2_ and HR responses to thigh WR loading during short-term submaximal running. It also gives means for quantifying an expected TLS for loaded submaximal running for a given duration. WRs attached to the legs enable a running-specific form of resistance training to be incorporated into training programing. Practitioners may be interested in moving the load distally away from the hip, as this placement seems to have a greater impact on metabolic cost, compared to commonly used trunk loading. However, this evidence is based only on 8 min of running and the effects of longer duration WR running under these conditions are still unknown and, more importantly, how the nature of this stimulus carries over to performance should be considered for future research. Building an understanding about how WR impacts the body acutely is important to help guide an evidence-based approach to programming, however, ultimately the potential longitudinal adaptations from WR require future investigations.

## Figures and Tables

**Table 1:** Acute oxygen responses with thigh wearable resistances.

Training Load (%BM)	Mean VO_2_ (L)	Effect Size (±90% CI)	Rating
0%	3.64 ± 0.57	-	-
1%	3.67 ± 0.59	0.13 (0.06; 0.21)	(7/93/0) likely trivial increase
2%	3.73 ± 0.62	0.13 (0.07; 0.19)	(3/97/0) very likely trivial increase
3%	3.80 ± 0.62	0.24 (0.17; 0.3)	(84/16/0) likely moderate increase
4%	3.84 ± 0.64	0.29 (0.2; 0.38)	(94/6/0) likely moderate increase
5%	3.94 ± 0.66	0.43 (0.37; 0.5)	(100/0/0) most likely very large increase

Abbreviations: BM, body mass; CI, Confidence interval. Values are the mean VO_2_ collected over the final 2 min period of 8 min of submaximal treadmill running at the first ventilatory threshold.

**Table 2:** Acute heart rate responses to thigh wearable resistance.

Training Load (%BM)	Mean Heart Rate (bpm)	Effect Size (±90% CI)	Rating
0%	157 ± 12	-	-
1%	157 ± 13	0.05 (−0.07; 0.16)	(2/98/0) very likely trivial increase
2%	159 ± 13	0.17 (0.02; 0.31)	(36/64/0) possible small increase
3%	159 ± 12	0.2 (0.07; 0.33)	(49/51/0) possible small increase
4%	161 ± 11	0.32 (0.16; 0.47)	(90/10/0) likely moderate increase
5%	161 ± 11	0.33 (0.21; 0.45)	(96/4/0) very likely large increase

Abbreviations: BM, body mass; CI, Confidence interval. Values represent the mean HR collected over the final 2 min period of 8 min of submaximal treadmill running at the first ventilatory threshold.

**Table 3:** Acute Lactate responses to thigh wearable resistances.

Training Load (%BM)	Mean LA (mmol/L)	Effect Size (±90% CI)	Rating
0%	2.62 ± 1.56	-	-
1%	2.77 ± 1.90	0.0 (−0.27; 0.28)	(12/77/11) unclear effect
2%	4.83 ± 2.04	0.08 (−0.07; 0.23)	(10/90/0) likely trivial increase
3%	3.27 ± 1.79	0.41 (0.23; 0.60)	(97/3/0) very likely large increase
4%	3.30 ± 2.03	0.42 (0.23; 0.61)	(97/3/0) very likely large increase
5%	3.52 ± 2.35	0.49 (0.34; 0.63)	(100/0/0) most likely very large increase

Abbreviations: BM, body mass; CI, Confidence interval. The values are mean blood LA accumulations sampled immediately post 8 min of submaximal treadmill running at the first ventilatory threshold.

**Table 4:** Acute rate of perceived exertion responses to thigh wearable resistances.

Training Load (%BM)	Mean Rate of Perceived Exertion	Effect Size (±90% CI)	Rating
0%	3.08 ± 1.37	-	-
1%	3.35 ± 1.16	0.28 (0.03; 0.53)	(70/30/0) possible small increase
2%	3.68 ± 1.44	0.43 (0.19; 0.66)	(95/5/0) likely moderate increase
3%	3.73 ± 1.33	0.52 (0.26; 0.78)	(98/2/0) very likely large increase
4%	4.20 ± 1.26	0.82 (0.53; 1.11)	(100/0/0) most likely very large increase
5%	4.38 ± 1.57	0.86 (0.58; 1.14)	(100/0/0) most likely very large increase

Abbreviations: BM, body mass; CI, Confidence interval. The values are mean RPE scores recorded immediately post 8 min of submaximal treadmill running at the first ventilatory threshold.
